# Cupula-Inspired Hyaluronic Acid-Based Hydrogel Encapsulation to Form Biomimetic MEMS Flow Sensors

**DOI:** 10.3390/s17081728

**Published:** 2017-07-28

**Authors:** Ajay Giri Prakash Kottapalli, Meghali Bora, Elgar Kanhere, Mohsen Asadnia, Jianmin Miao, Michael S. Triantafyllou

**Affiliations:** 1 Center for Environmental Sensing and Modeling (CENSAM) IRG, Singapore-MIT Alliance for Research and Technology (SMART), 1 Create Way, Create Tower, Singapore 138602, Singapore; meghali@smart.mit.edu; 2 School of Mechanical and Aerospace Engineering, Nanyang Technological University, 50 Nanyang Avenue, Singapore 639798, Singapore; elgarvik001@e.ntu.edu.sg (E.K.); mjmmiao@ntu.edu.sg (J.M.); 3 Department of Engineering, Macquarie University, Sydney NSW 2109, Australia; mohsen.asadnia@mq.edu.au; 4 Department of Mechanical Engineering, Massachusetts Institute of Technology (MIT), 77 Massachusetts Avenue, Cambridge, MA 02139, USA; mistetri@mit.edu

**Keywords:** biomimetic sensors, hydrogels, microelectromechanical systems, flow sensing

## Abstract

Blind cavefishes are known to detect objects through hydrodynamic vision enabled by arrays of biological flow sensors called neuromasts. This work demonstrates the development of a MEMS artificial neuromast sensor that features a 3D polymer hair cell that extends into the ambient flow. The hair cell is monolithically fabricated at the center of a 2 μm thick silicon membrane that is photo-patterned with a full-bridge bias circuit. Ambient flow variations exert a drag force on the hair cell, which causes a displacement of the sensing membrane. This in turn leads to the resistance imbalance in the bridge circuit generating a voltage output. Inspired by the biological neuromast, a biomimetic synthetic hydrogel cupula is incorporated on the hair cell. The morphology, swelling behavior, porosity and mechanical properties of the hyaluronic acid hydrogel are characterized through rheology and nanoindentation techniques. The sensitivity enhancement in the sensor output due to the material and mechanical contributions of the micro-porous hydrogel cupula is investigated through experiments.

## 1. Introduction

Biomimetic sensor development is an interdisciplinary field involving material science, biology and micro/nano engineering, in which, inspirations from biology are mimicked in developing novel sensor designs, sensing principles and for investigating new materials for sensors. It involves the investigation of both structural and material designs of the biological counterparts of interest with the goal of engineering artificial sensors [1]. Biological organisms feature a number of sensory systems which work on micro/nano mechanical principles such as hairs in humans which perform tactile sensing [2], vibration detection through shaft-like bending of hairs in spiders [3], flexible membranes for night vision in snakes [4], undulated whiskers for wake tracking in seals [5], and neuromasts for navigation in fishes [6]. This work is inspired from the neuromast sensors on the lateral-line of fishes, which help them to locate and identify their predator, prey and surrounding objects as well as aid in efficient maneuvering.

## 2. Bioinspiration

**Figure 1:**
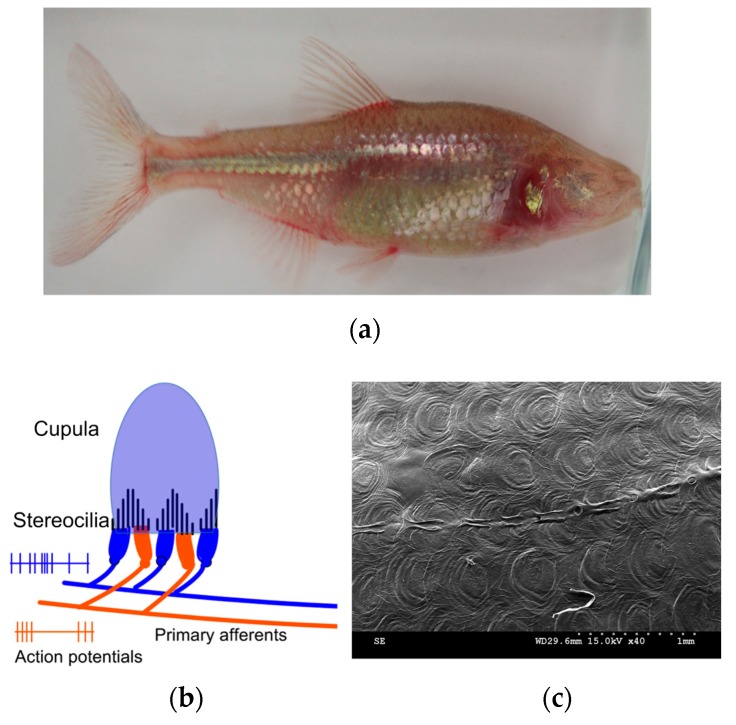
Bioinspiration—biological neuromast flow sensors in blind cavefish: (**a**) A photograph of a blind cave tetra which shows the regressed eyes and pigmentation due to dwelling in dark deep caves; (**b**) A schematic showing the morphology of the biological neuromast sensor, which includes the cupula and the cilia; (**c**) A scanning electron microscope (SEM) image of the lateral-line of the blind cave fish that runs across the length of the fish on the sides.

The blind cave fish (*Astyanax mexicanus*, Figure 1a)*,* also called the blind cave tetra, is capable of generating a hydrodynamic view of its surrounding environment. It thereby adeptly swims through underwater obstacles while finding its way around by means of lateral-lines, which are highly sensitive to ambient flows. The sensory organs in fishes consist of two types of sensors, which are spread all over the surface of the fish body. Superficial neuromasts (SN) that are located on the surface of the skin (Figure 1b), and canal neuromasts (CN) (Figure 1c) that are embedded inside the microfluidic channels called canals [6,7,8]. Each neuromast sensor consists of haircells that are embedded into a soft material called cupula. The cupulae are made up of gelatinous glycol protein material. They consist of glycosaminoglycan gel material that is transparent and extremely soft with orders of magnitude lower Young’s modulus than the embedded cilia. The cupula acts as a mechanical structure that captures any relative motion between the fish and its surrounding water [9]. The cupular material has nearly the same density as the surrounding water and therefore is hypothesized to be driven mainly by viscous forces [10]. The cupulae enhance the drag force on the haircells in several ways. The cupula increases the overall surface area of the neuromasts as compared to not having it i.e. sensing by the bare haircells. Increased drag force causes increased bending of the haircells generating a better signal and enhances the signal transmission to the haircells [9,11]. It is hypothesized in the past that the hydrogel-like material that makes up the cupula has a hydrophilicity and permeability that enhances the signal absorption through an enhanced friction factor associated with the material [12].

In the past, a few groups have worked towards developing microelectromechanical systems (MEMS) sensors for underwater sensing applications inspired from the biological lateral-line of fish. The complexity of the biological neuromast has inspired researchers to explore various aspects in the design of the biomimetic flow sensors, such as materials, design, structure, sensing principle etc. Engel et al. have described a method of fabricating an all-polymer artificial hair cell sensor utilizing polyurethane material to form the hair cell structure and the force sensitive resistors (FSRs) which form the sensing elements of the sensor [13]. Flexible and conductive FSRs are achieved by loading polyurethane with conductive fillers such as carbon black and carbon multi-walled nanotubes. Two axis sensing of flows was made possible by arranging the FSRs in half-bridge configuration. Kottapalli et al. have developed their artificial MEMS neuromast flow sensors utilizing a liquid crystal polymer membrane material [14,15,16,17]. Their sensor featured 3D printed high-aspect ratio polycarbonate hair cells that extend beyond the flow-generated boundary layers and enhance the flow sensitivity. Inspired by the neuromast, Chen et al. have fabricated ultrasensitive MEMS flow sensors that feature SU-8 hair cells on thin paddle-shaped silicon cantilever beams and are capable of measuring oscillatory flow velocities as low as 1 mm/s [18]. They have also experimentally demonstrated that their sensors were capable of resolving directionality of flows up to 2.16°.

Researchers have also explored various sensing principles in the development of MEMS artificial hair cell sensors. Piezoresistive elements both in the form of strain gauges and ion implanted resistors have been implemented at the base of the hair cell [19,20,21,22,23]. The torque generated due to the bending of the hair cell causes a change in resistance in the piezoresistor. Asadnia et al. have instead utilized piezoelectric thin film Pb(Zr)_0.52_(Ti)_0.48_ (PZT) membranes as sensing elements in their silicon based MEMS hair cell sensors [24]. These sensors feature the advantage of being inherently self-powered and can reject static and steady flow generated sensor outputs [25]. While the piezoresistive sensors developed by Kottapalli et al. were experimentally tested for steady-state flows, the piezoelectric sensors developed by Asadnia et al. were more suitable for oscillatory flows [19,20,21,22,23,24,25,26,27]. Dijkstra et al. utilized capacitive sensing methods to detect the movement of the hair cell in their biomimetic cricket receptor inspired hair cell sensors [28]. A silicon-nitride membrane that is attached to the base of the hair features two electrodes that distinguish between rotation and translation normal to the substrate. Klein et al. developed artificial lateral line canal sensors which featured hair cell sensors packaged into cylindrical canals and exposed to external flows through pores on the canals which work through optical sensing principles [29]. They used transparent silicone bars as hair cells and the bending of the hair cell was determined through the light transmitted through the hair cell. Abdulsadda et al. have utilized novel sensing materials such as ionic polymer metal composites (IPMC) for fabricating the artificial MEMS sensors [30]. Application of IPMC material brings these sensors some advantages such as capability to measure flow polarity, simple sensor fabrication and high sensitivity. Amongst the recent works Yilmazoglu et al. reported a novel artifical hair cell sensor which utilizes three-dimensional vertically aligned carbon nanotube bundles [31]. Their CNT sensors are capable of stable mechanical bending upto 90° and can detect hair cell bending as low as 1 μm. In other works interesting artificial hair cell structures such as stress-driven out-of-plane bent cantilever beams have been fabricated utilizing the inherent stress between multilayers in the cantilever [32]. Yang et al. have used hot wire anemometry based sensing to measure oscillatory flow velocities [33]. They demonstrated novel fabrication methods such as 3D magnetic assembly to form unique out-of-plane hair cell design with sensing elements elevated into the flow. In a pursuit to enhance the sensitivity of the MEMS hair cell flow sensors, some researchers took a biomimetic material approach and implemented artificial cupula-like materials on the hair cell and demonstrated an enhancement in the sensitivity due to the presence of the cupula material [19,20,23,24]. A few researchers have also developed arrays of hair cell sensors to form an artificial lateral line which has immense applications in object detection, artificial hydrodynamic vision and control of underwater robots [29,30,33,34,35]. Comprehensive reviews of various artificial MEMS hair cell sensors and their sensing abilities is provided in [36,37,38].

This work presents the design, fabrication and experimental testing of a MEMS artificial neuromast sensor that features a high aspect ratio SU-8 hair cell and a hydrogel cupula that encapsulates the hair cell. In contrast to the piezoresistive hair cell sensors developed in the past, these sensors feature the entire full-bridge circuit on the sensing membrane, which not only enhances the sensitivity of the sensor by four times as compared to quarter bridge circuits but also eliminates the need for any external circuits and compensates for temperature variations [39]. This work also proposes a fabrication method that monolithically integrates the 3D hair cell structure development with the fabrication of the sensing membrane and the device dicing. In addition, the work reports the synthesis and application of a hyaluronic acid (HA) hydrogel cupula material whose material properties closely match with those of the biological cupula in the superficial neuromasts.

## 3. MEMS Artificial Neuromast

### 3.1. Sensor Structure and Sensing Principle

**Figure 2:**
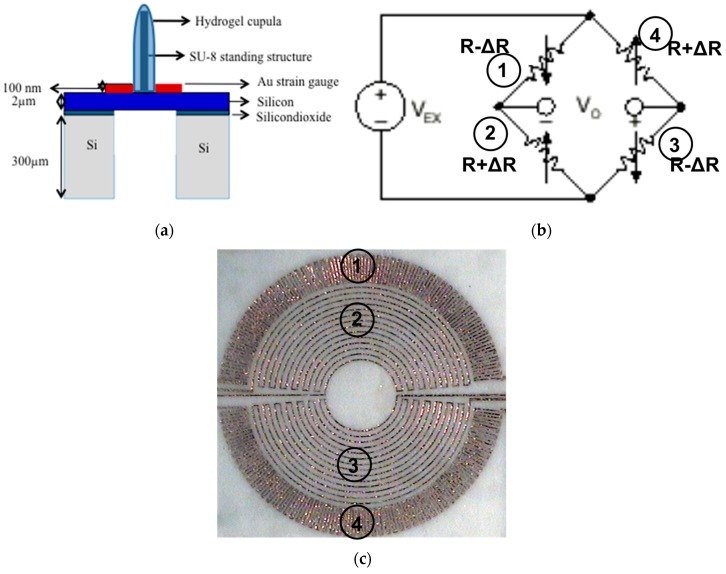
Artificial MEMS neuromast sensor structure and design: (**a**) Schematic showing the sensor structure; (**b**) Circuit diagram that describes the full-bridge connection of the resistors on the membrane; (**c**) An optical image of the fabricated sensor showing the positioning and geometry of the two radial and the two spiral strain gauges.

The sensor structure consists of three main parts—the sensing membrane with full bridge strain gauges, the SU-8 hair cell and the hydrogel cupula. The silicon sensing membrane of 1500 μm diameter and 2 μm thickness features an SU-8 hair cell, 600 μm tall and 150 **μ**m in diameter that extends from the center of the silicon membrane. A full Wheatstone bridge circuit consisting of two radial and two tangential resistors is fabricated on the membrane. A HA hydrogel cupula is drop-casted on the hair cell which encapsulates the hair cell completely. A schematic of the sensor structure is show in Figure 2a.

Disturbances in the flow exert a drag force on the hydrogel cupula which inturn transduces the force to the embedded hair cell causing it to deflect. Since the SU-8 hair cell is attached to the silicon membrane at the base, the deflection of the hair cell generates a displacement in the membrane. The membrane displacement causes resistance changes in the four resistors. The resistance change is converted to a voltage change by biasing the full bridge circuit with a voltage of 5 V.

### 3.2. Design

One of the key design aspects for the proposed biomimetic MEMS flow sensor is to ensure that the positioning and geometry of the strain gauges allow maximum sensitivity of the sensor. The right positioning of the strain gauges depend on three main factors—geometry of the membrane, boundary conditions and the type of load [39]. In order to maximize the resistance values while maintaining a smaller membrane size, rosette and spiral patterns covering a considerable area of the membrane are designed (Figure 2b,c). For the radial strain gauges, a rosette design was placed at the rim of the membrane where the maximum strain occurs (Figure 2c). The gold joints linking two radial joints of the rosette were made shorter and wider since they are strained perpendicular to the electric current and would negate the overall signal [39]. The spiral strain gauges, which collect the tangential strain, are placed next to the rosette at the center of the membrane. The radial and the spiral strain gauges form the two half of the entire Wheatstone bridge (Figure 2b,c). The full-bridge on membrane design ensures that the four strain gauges are positioned at areas that experience different strain but same temperature, thereby compensating for the drift effects that temperature changes would cause. All the four resistors are designed to have the same resistance value so that the absence of external flow stimuli would result in to zero output voltage from the sensor. In the presence of external flow, the membrane bends causing an imbalance of the resistance values and thereby a voltage output corresponding to the flow velocity is generated.

**Figure 3:**
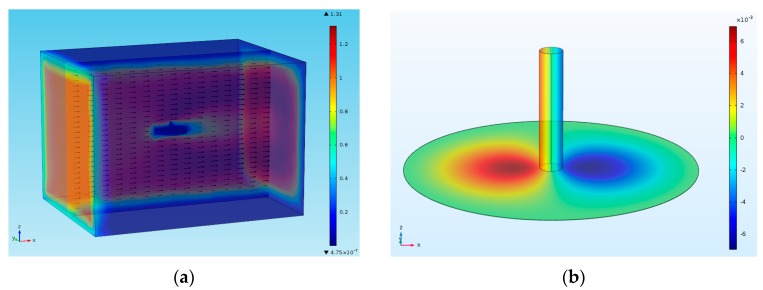

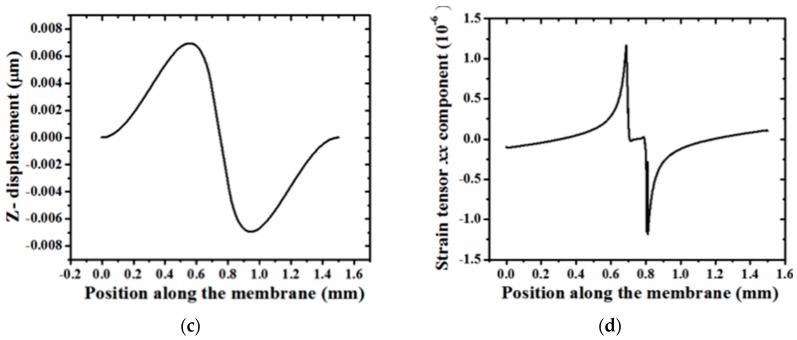
Three-dimensional finite element analysis structural mechanical simulations to determine the stress distribution on the membrane: (**a**) Simulation set-up describing the sensor structure subject to air flow velocity of 1 m/s (hair cell sensor geometry and material parameters set to real case); (**b**) Displacement mapping on the LCP membrane; (**c**) Displacement profile along the central axis (diameter) of the membrane; (**d**) Strain distribution along the membrane.

Three dimensional finite element structural analysis simulations have been conducted to obtain the displacement profiles of the sensing membrane and the strain distribution on the membrane for various experimental flow velocities. A three-dimensional structure of the silicon membrane hinged at the circumference with a rigid pillar at the center was used for the simulation (Figure 3a). In the structural mechanical simulation, the geometry and the material parameters of the device have been set to be the same as in the MEMS sensor. The geometry includes a device with a diaphragm with thickness of 2 μm and diameter of 1500 μm. A cavity of 1500 μm diameter and 0.4 μm depth is present beneath the diaphragm. A pillar of 150 μm diameter and 600 μm height is mounted at the center of the diaphragm. Fixed constraints are applied on the circumference of the circular diaphragm and the body of the device. The hair cell was defined to be fixed at the root and free to move at its distal tip. A pressure of 3 Pa, which is approximately equal to the pressure that would be exerted by an air flow of 1 m/s, is applied as a boundary load in positive X-direction on the standing pillar. Figure 3c,d show the surface plots depicting *Z*-component of displacement of diaphragm and *XX* component of strain tensor distribution across the diaphragm.

### 3.3. Fabrication

The device fabrication mainly consists of four major steps: (1) gold sputtering and lift-off, (2) spin-coating of a thick (600 µm) SU-8 2150 layer, (3) DRIE through-hole etching from backside to simultaneously release the device membrane and perform dicing, (4) SU-8 exposure and resist-developing to form high aspect ratio pillars. Since the device consists of standing pillars of high aspect ratio, conventional mechanical dicing using diamond wheel cannot be performed. It was found that the standing pillars were repeatedly uprooted due to high vibration created by the mechanical diamond wheel during dicing. Also, the water flow jet used for cooling the wheel during the mechanical dicing process can cause the pillars to detach from the membrane. Therefore, we have developed a fabrication procedure that not only monolithically integrates the fabrication of the sensing membrane, the strain gauges and the 3D SU-8 hair cell, but also performs the dicing of the devices alongside with the membrane release process.

**Figure 4:**
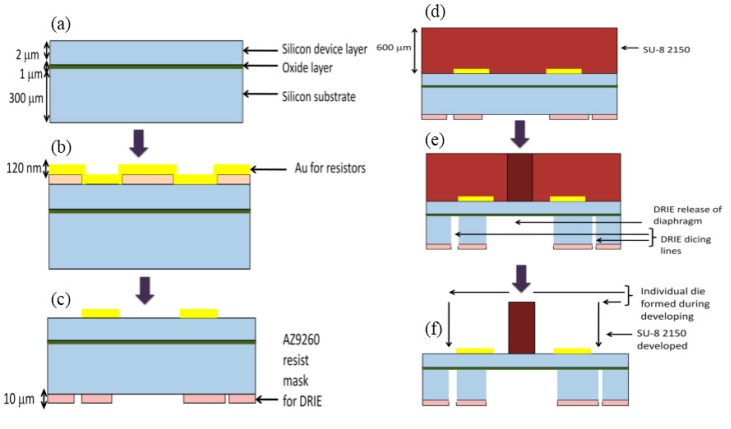
Biomimetic sensor fabrication: (**a**) SOI wafer structure; (**b**) Resist patterning followed by gold sputtering; (**c**) Lift-off process and backside resist patterning for DRIE mask; (**d**) SU-8 2150 spin-coated on top side; (**e**) SU-8 patterning and DRIE through-holes on backside of wafer; (**f**) SU-8 developing to form standing pillar.

The steps involved in the fabrication scheme is illustrated pictorially in Figure 4. The fabrication process uses a silicon on insulator (SOI) wafer with 2 μm thick device layer and 300 μm thick handle layer as shown in Figure 4a. Initial piranha cleaning of the wafer is followed by baking for 20 min, at 130 °C, to make the surface hydrophilic. After performing 1 min of hexamethyldisilazane (HMDS), a 5 µm thick AZ-9260 positive photoresist (PR) is spin-coated and patterned using a glass mask for lift-off as shown in Figure 4b. A 20 nm/120 nm thick Cr/Au layer is deposited by sputtering process as shown in Figure 4b. This layer forms the gold resistors, which act as a strain gauge sensing elements. A thin layer of gold is deposited to increase the resistance of individual resistors. After this, a lift-off process is performed by immersing the wafer in acetone. The device structure after the lift-off process is shown in Figure 4c. The next step is to spin-coat and pattern on the back side of the wafer with a PR resist mask for the DRIE process. A 10 µm thick AZ-9260 PR is spin-coated and patterned while aligning the mask carefully to the strain gauge resistors on the front side as shown in Figure 4c. This PR mask was designed to define the membrane release as well as dicing lines for the DRIE process.

The next step is spin-coating a thick layer of SU-8 2150 on the front side (the side with gold resistors) to form the standing pillar. The wafer is baked on a hotplate for 20 min at 100 °C before spin-coating SU-8. This step will harden the PR on the back side and will ensure that it acts as a good mask for DRIE and also, make the PR hard enough to survive further front side SU-8 processing. A thick layer of SU-8 2150 is spin-coated after 1 min of HMDS as shown in Figure 4d. A 10 mL quantity of SU-8 2150 is dispensed and left for about 5 min to spread by itself due to gravity. After the SU-8 spread to three-fourths of the wafer area, the spinning process is performed. Spin-coating is done at 500 rpm for 10 s with an acceleration of 100 rpm/s and followed by 1000 rpm for 35 s with an acceleration of 300 rpm/s. After spin-coating, the wafer is transferred to a hotplate for the prebaking process. The prebaking process is crucial because it evaporates the solvent from the SU-8 layer allowing it to harden. The prebake is done at 65 °C for 30 min, and then the temperature is ramped up to 95 °C and maintained there for 150 min. It is important to conduct all the baking processes for prolonged times to avoid inbuilt stresses in the thick SU-8 layer which will affect the shape of the pillars after exposure and developing process. Moreover, temperature changes during baking must be conducted gradually and not in big steps to avoid wafer buckling and non-uniformity of SU-8 thickness over the wafer surface. After prebaking, the wafer is removed from hotplate and is allowed to cool down to room temperature.

**Figure 5:**
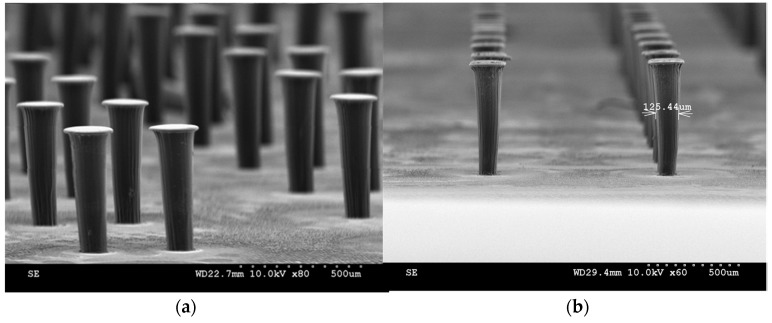
SU-8 3D hair cell structure fabrication: (**a**,**b**) show SEM images of arrays of SU-8 hair cells.

The SU-8 layer is exposed for 72.6 s during lithography. However, the exposure time calculated from the SU-8 2150 data sheet was 69.9 s, but the value of 72.6 s is concluded to be the best after a lot of optimization. SU-8 is a negative photoresist, and the exposed regions get hardened, and remain during developing, forming pillar-like structures through the depth of the layer as shown in Figure 4e. The SU-8 process to form standing structures is optimized on bare silicon wafers by spin-coating, followed by prebaking, exposing and post-baking. After developing, the pillars are observed in a microscope to verify that the development process is complete. All the baking times and temperatures, exposure time and developing time are optimized on bare dummy silicon wafers before trying on an actual wafer. SEM images of SU-8 standing pillar arrays are shown in Figure 5.

**Figure 6:**
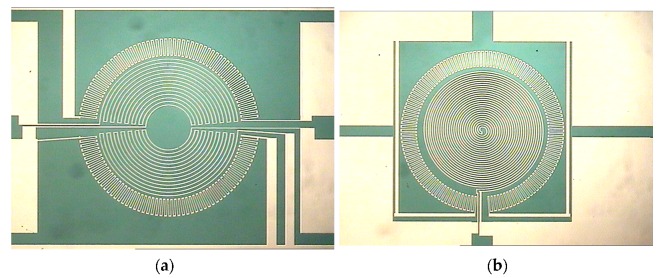

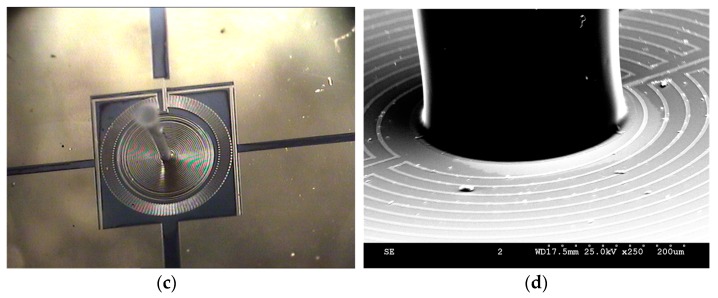
SU-8 hair cell sensor: (**a**) Full bridge on 2 μm silicon membrane showing two radial and two spiral resistors; (**b**) Half bridge on 2 μm silicon membrane showing one radial and one spiral resistor; (**c**) SU-8 hair cell fabricated at the center of the strain gauges; (**d**) A SEM image showing the root of the SU-8 hair cell.

The SU-8 layer is post-baked at 65 °C for 15 min, and then the hotplate is ramped up to 95 °C and maintained for 60 min. The post-baking process is crucial to harden the mask and make it suitable for prolonged developing process. However, the developing process is not conducted soon after post-bake, but is conducted after the DRIE process. This is made feasible by not allowing the SU-8 layer to be exposed to white light at any time until the DRIE process is completed. This is ensured by sticking a support wafer onto the SU-8 processed side of the main wafer. The support wafer helps to accomplish the DRIE process, as well as, to protect the SU-8 layer from any exposure before developing. A 300 µm deep DRIE through-hole etch is conducted which is etch-stopped at the 1 µm oxide layer. A schematic of device structure after DRIE is as shown in Figure 4f. After the DRIE process, the wafer-pair is kept immersed in acetone for 3 days to detach the support wafer. While doing this process, the wafer-pair is kept enclosed in a box, sealed in a black cover to avoid any exposure. Although individual device dicing is already performed during the DRIE step, the thick SU-8 layer still supports the wafer. The wafer is immersed in SU-8 2150 commercial developer for 90 min. A very slight agitation is done while developing, because as developing progresses, the standing pillars start appearing, and an external agitation can cause the pillars to bend or even get uprooted and therefore it is concluded to conduct developing at very low mechanical agitation. The developing process is conducted for a prolonged time duration of 90 min since no agitation is done during developing. As the developing process proceeds, the SU-8 layer becomes thinner and thinner, and at a point, the devices get diced automatically due to a very thin device layer (2 µm thick) holding them together. After the developing process, the individual dies are carefully collected, placed in isopropyl alcohol (IPA) and followed by rinsing in water. The optical and SEM images of the fabricated half-bridge and full-bridge devices with SU-8 hair cell is shown in Figure 6.

## 4. Biomimetic Hydrogel Cupula

### 4.1. Synthesis of HA Hydrogel Cupula

HA was first modified using methacrylic anhydride (MA) before synthesizing the hydrogel as described previously [40]. HA was dissolved in deionized (DI) water to get 1.5% *w*/*v* solution and its pH was adjusted to 8 using 1 M NaOH. Around 20 molar excess of MA was added to HA solution and pH was maintained at 8. The reaction was allowed to continue for 2 h and then the solution was kept at 4 °C fridge for 24 h. The modified HA (HA-MA) solution was dialyzed extensively using NaCl, ethanol, and ultrapure water and then lyophilized for 72 h before using it for drop casting over SU-8 hair cell.

### 4.2. Encapsulation Technique

Once the HA-MA was completely dried, it was dissolved in DI water to get a concentration of 2% *w*/*v*. 0.1% *w*/*w* solution of a photoinitiator (Irgacure 2959 was prepared in 70% *v*/*v* ethanol and mixed with the HA-MA solution thoroughly). This HA-MA solution was carefully drop cast over SU-8 hair cell with the volume added being just sufficient to cover the entire hair cell along its length and bottom without crossing beyond its circumference. It was then exposed to UV light at 365 nm for 10 min for crosslinking. The crosslinked HA-MA gel was swollen in DI water at room temperature (RT) for 24 h.

### 4.3. Hydrogel Characterization

**Figure 7:**
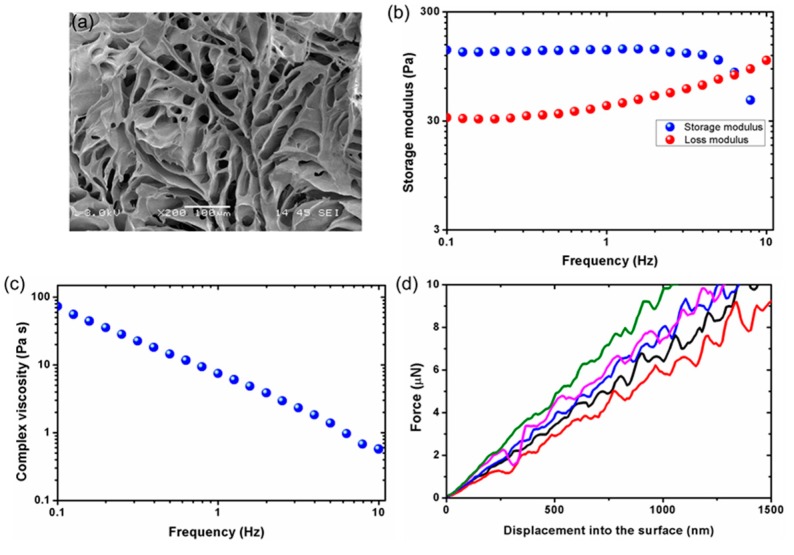
Characterization of 2% HA-MA hydrogel: (**a**) SEM image of a cross-section of dried hydrogel sample showing the network of pores; (**b**) Frequency sweep plot showing the constant stiffness of hydrogel independent of frequency range up to 3 Hz; (**c**) Complex viscosity plot showing shear thinning behavior with increasing frequency; (**d**) Nanoindentation plot showing force-displacement curves for five hydrogel surface locations.

HA-MA hydrogel was characterized for morphology, swelling behavior, and mechanical properties. Figure 7a shows a SEM micrograph of a cross-section of 2% hydrogel showing its porous mesh network. For swelling study, wet and dry mass of hydrogel were recorded before and after swelling and used to calculate swelling ratio, water content, number of crosslinks per unit volume (crosslinking density), and mesh size. Table 1 below shows the approximate values of these parameters calculated using Flory-Rehner equations [41].

Rheological and nanoindentation analysis of 2% water swollen HA-MA hydrogel were conducted to understand their mechanical properties. For rheology, a stress controlled rheometer (Physica MCR 501, Anton Parr, Ashland, VA, USA) was used with a measuring system of parallel plate geometry. The hydrogels were subjected to an oscillatory shear test with a frequency sweep (0.1 to 10 Hz) at a fixed strain rate while the storage modulus (G**′**), loss modulus (G**″**), and complex viscosity values were obtained. Figure 7b shows the stiffness of HA-MA hydrogels, with the G**′** being constant up to 3 Hz and then starting to decrease slowly with increase in frequency. There was a significant difference between G**′** and G**″** for most part of the frequency range tested. G**″** increased gradually after around 0.7 Hz crossing G**′** around 8 Hz. At 1 Hz, G**′** was around 162 Pa corresponding to a Young’s modulus (E**′**) of around 5 Pa [42]. Figure 7c shows the complex viscosity decreasing linearly with frequency. This is representative of the shear thinning of hydrogels that results from the phenomenon related to the dynamics of mechanical energy dissipation in entangled chains of hydrogel network. For nanoindentation, a G200 nanoindenter (Agilent Technologies, Singapore) machine was used with a diamond cono-spherical tip (apex radius of 5 µm) to calculate E**′**. The indentation was conducted at high-resolution mode with the tip set to ultrasensitive mode and speed of tip approach set to 20 nm/s. Figure 7d shows a plot of force exerted on the hydrogel surface against the displacement observed during the unloading process. It shows the results of five indentations performed over five different locations across the HA-MA hydrogel. The average E′ obtained from the force-displacement curve was found to be around 8 Pa which is similar to the biological cupula.

The rigid yet porous structure of the HA hydrogel cupula improves the sensitivity of the MEMS sensor by enhancing the fluid motion induced viscous drag forces [12,19]. Besides these properties, the hydrogel cupula increases the overall surface area exposed to the incoming flow stimulus, which increases the drag force thus enhancing the sensitivity. The viscous coupling of the cupula and the very low relaxation times reduce the random noises, low frequency noises, and diminish the effects of Brownian motion, thereby enhancing the signal to noise ratio of the sensor [19,23,24,42]. Therefore, in order to improve the sensitivity of the sensor, it is very important to understand the morphological and material properties of the HA-MA hydrogel. Increased surface area, hydrophilicity of the polymer material, friction component due to interaction of surrounding water with the water trapped in pores of hydrogel matrix could contribute towards the enhancement of viscous drag forces and thereby the sensitivity [12].

## 5. Experimental Results

We have conducted proof-of-concept experiments to determine the flow sensing performance of the biomimetic MEMS sensor. Both the naked hair cell sensor and the hydrogel-capped sensors were tested to investigate the enhancement in the sensing performance of the sensor due to the biomimetic cupula-inspired hydrogel capping. The full-bridge circuit was biased with a DC voltage of 5V and the output from the sensor is connected to a National Instruments data acquisition card (NI-DAQ, USB-6289 M-series model, National Instrument, Austin, TX, USA) and is recorded using the Signal Express module of the LABVIEW software.

**Figure 8:**
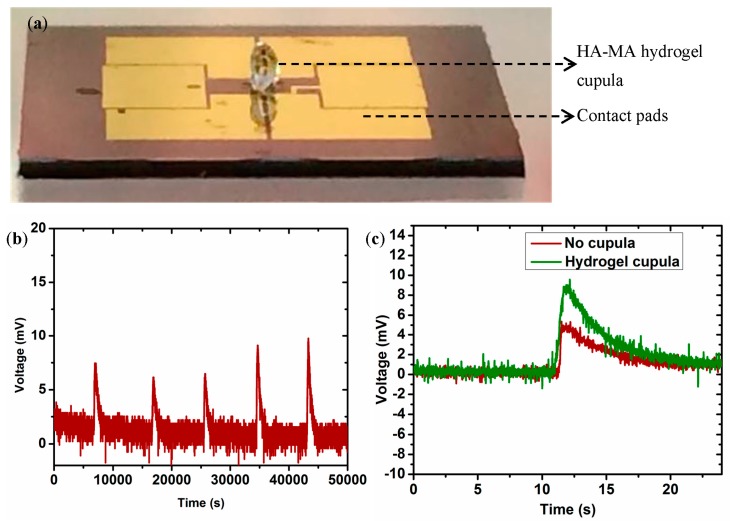
Experimental flow sensing results: (**a**) MEMS sensor with biomimetic hydrogel capping on the hair cell; (**b**) Response of the naked hair cell sensor to five pulses of air flow of velocity 0.2 m/s; (**c**) Comparison of the output of the hydrogel-capped sensor with respect to that of the naked hair cell sensor in response to a flow velocity of 0.1 m/s.

Figure 8b shows the output of the naked hair cell sensor for a stimulus of five pulses of airflow past the sensor. As it can be seen, the sensor produced five distinct and well-defined peaks at the time instants when the air flow past the hair cell. In another experiment, in order to investigate the improvement in the sensitivity of the sensor due to the presence of the hydrogel capping, both the naked hair cell (SU-8 hair cell) sensor and the hydrogel-capped sensor were placed in the presence of a flow velocity of 0.1 m/s. Figure 8c shows the output of both these sensors. It can be seen that the hydrogel-capped sensor showed a much higher output voltage in response to the flow. The presence of the cupula increases the overall surface area exposed to the flow and thereby enhances the drag force experienced by the structure offering additional sensitivity to the sensor. The HA-MA hydrogel used is highly absorbant with 85% water in them and also a similar density to water. In addition, the porosity and hydrophilicity of hydrogels can help in increasing the signal transmission from the flow to the embedded hair cell. There exists a coupling interaction between the external flow and the water trapped in the loose semi-permeable pores of hydrogel matrix, thus increasing the hydrophilicity increases the drag force. Therefore, the presence of the cupula offers both mechanical and material contributions towards the enhancement of sensitivity of the sensor.

## 6. Discussion and Conclusions

This work develops a MEMS flow sensor which features a 3D cylindrical hair cell inspired by the biological neuromast sensor present in the blind cavefish. The MEMS sensor consists of a 2 μm thick silicon sensing membrane, which consists of gold strain gauges patterned and connected into a full-bridge circuit. As compared to the bioinspired hair cell sensors developed in the past, this sensor features three key advantages. First, the sensor design eliminates the need for an external Wheatstone bridge circuit since the full-bridge circuit is achieved on the sensing membrane itself. Second, full-bridge circuit on membrane as compared to the quarter bridge circuits developed in the past, allow a four times higher sensitivity. Third, the fabrication method monolithically integrates the 3D hair cell fabrication along with the fabrication of the MEMS sensing membrane and the dicing process. A biomimetic artificial cupula is synthesized using Hyaluronic acid hydrogel and drop-casted on the SU-8 hair cell. The presence of the artificial HA hydrogel cupula enhances the sensitivity of the sensor both due to mechanical and material contributions of the hydrogel. The hydrogel cupula increases the cross-sectional surface area exposed to flow as compared to the SU-8 hair cell and thereby increases the drag force experienced by the standing structure. Increased drag force in the presence of the cupula causes an enhanced bending moment on the membrane and a higher sensor output. In addition, the porous structural pockets of HA hydrogel filled with water and material permeability seem to have contributions in further enhancing the drag force. The paper describes in detail the design, fabrication and experimental characterization of the flow sensor. The methodology and fabrication techniques demonstrated in this work could guide the development of further biomimetic materials and sensors in future. Flexible arrays of such MEMS hair cell sensors representing the lateral-line in fish could be utilized on underwater vehicles to attain hydrodynamic vision and energy-efficient maneuverability that fishes naturally achieve.

## Figures and Tables

**Table 1:** Mesh structure parameters of 2 HA-MA hydrogels.

Parameter	Value
Swelling ratio	37.4
Equilibrium water content	85%
Crosslinking density	2.5 × 10^−6^ mol/cm^3^
Mesh size	433 nm
